# The role of protease-activated receptors (PARs) in the functioning of platelets and platelet-derived microparticles (PMPs)

**DOI:** 10.3389/fmolb.2025.1636893

**Published:** 2025-09-09

**Authors:** Urszula Jakobsche-Policht, Agnieszka Bronowicka-Szydełko, Rajmund Adamiec, Dorota Bednarska-Chabowska, Magdalena Mierzchała-Pasierb, Łukasz Lewandowski, Kinga Gostomska-Pampuch, Joanna Adamiec-Mroczek, Maciej Rabczyński, Edwin Kuźnik, Paweł Lubieniecki, Olgierd Dróżdż, Helena Martynowicz, Anna Kwiecień, Małgorzata Strzelecka, Dawid Rudkiewicz, Marcin Piersiak, Maciej Ziomek, Mikołaj Kondracki, Zuzanna Galińska, Katarzyna Madziarska

**Affiliations:** ^1^ Department of Angiology and Internal Diseases, Institute of Internal Diseases, Wroclaw Medical University, Wroclaw, Poland; ^2^ Department of Biochemistry and Immunochemistry, Wroclaw Medical University, Wroclaw, Poland; ^3^ Clinical Department of Diabetology, Hypertension and Internal Diseases, Institute of Internal Diseases, Wroclaw Medical University, Wroclaw, Poland; ^4^ Clinical Department of Ophthalmology, Wroclaw Medical University, Wroclaw, Poland; ^5^ Department of Basic Chemical Sciences, Wroclaw Medical University, Wroclaw, Poland; ^6^ Department of Medicinal Chemistry, Wroclaw Medical University, Wroclaw, Poland; ^7^ Faculty of Medicine, Wroclaw Medical University, Wroclaw Medical University, Wroclaw, Poland

**Keywords:** atherosclerosis, hemostasis, platelet-derived microparticles, protease-activated receptor (PAR-1), vessels

## Abstract

Protease-activated receptors (PARs), present on the surface of platelets and platelet-derived microparticles (PMPs), belong to a superfamily of membrane receptors that play a key role in initiating intracellular G protein-dependent signaling pathways. Although four types of PARs have been identified–PAR-1, PAR-2, PAR-3, and PAR-4 – their mechanisms and functions remain poorly understood. Nevertheless, they are considered promising therapeutic and diagnostic targets, as they play crucial roles in initiating and promoting processes such as coagulation, inflammatory responses, and vascular function. PAR-1 is expressed on various cell types, including endothelial cells, platelets, neurons, and immune cells. Its activation by thrombin initiates a G protein-dependent signaling cascade that stimulates the expression of cytokines, selectins, adhesion molecules, and growth factors. In addition to thrombin, PAR-1 can also be activated by activated protein C (APC) and matrix metalloproteinase-1 (MMP-1). APC triggers cytoprotective signaling pathways, while MMP-1 influences cellular dynamics through alternative signaling mechanisms. PAR-1 activation is also affected by epigenetic modifications and genetic polymorphisms in the PAR-1 gene. Variants such as −1426 C/T and −506 I/D influence receptor expression and are associated with an increased risk of thrombosis, potentially due to epigenetic changes linked to atherosclerosis. The complex signaling network of PAR-1 makes it a promising therapeutic target for the treatment of cardiovascular diseases, cancer, and neuroinflammatory disorders. This paper serves as a compendium on PAR-1 and its role, particularly in the activation of platelets and PMPs.

## 1 Introduction

Hemostasis is a series of processes aimed at maintaining blood in a fluid state within the vascular system and preventing its extravasation following vessel injury through the formation of a platelet plug, which is subsequently transformed into a fibrin clot. Key components of hemostasis include: platelets (PLTs), the blood vessel wall, the plasma coagulation system, endogenous anticoagulant mechanisms, and the fibrinolytic system. An important factor is also the flow dynamics of circulating blood (Windyga J and Undas A., 2014). Hemostasis most commonly occurs through the simultaneous processes of coagulation and fibrinolysis, which remain in balance with each other. The dominance of one process is the result of the prevailing activity of its enzymes over the enzymatic activity of the opposing process (Zawilska K., 2019).

The progression of atherosclerosis depends, among other factors, on epigenetic changes occurring in: endothelial cells, vascular smooth muscle cells, immune cells (monocytes, macrophages, lymphocytes), stem and progenitor cells, as well as in blood platelets and platelet-derived microparticles (PMPs) (Mehu et al., 2022). These changes can occur at various levels, including DNA methylation (e.g., increased methylation of promoters of anti-inflammatory and protective genes such as *KLF4* and *ABCA1* may reduce their expression), or histone modifications (e.g., acetylation, methylation, or phosphorylation of histones such as H3K4me3, H3K9me2, and H3K27me3, which affect chromatin structure and DNA accessibility for transcription factors) (Wu et al., 2023). Non-coding RNAs (primarily microRNAs) also play a significant role, as they bind to mRNA and regulate its stability and translation (Nemeth et al., 2024). Epigenetic changes in chromatin can lead to its long-term reorganization, affecting “cellular memory” in vascular cells, for example by sustaining a pro-inflammatory state even after the triggering factor has been removed (Hu et al., 2012).

The mechanisms underlying atherosclerosis regression in blood vessels aim to: 1) protect endothelial function and improve its integrity by limiting glycocalyx damage, increasing nitric oxide (NO) bioavailability, inhibiting oxidative stress (NADPH oxidase, ROS), and stabilizing intercellular junctions (PECAM-1, VE-cadherin, Hippo-YAP/TAZ pathway); 2) reduce chronic inflammation by inhibiting pro-inflammatory cytokines (IL-1β, TNF-α), interrupting positive feedback loops of inflammation, and regulating epigenetic factors such as microRNAs, DNA methylation, and histone modifications; 3) modulate vascular smooth muscle cell (VSMC) phenotype by preventing transition to synthetic/osteochondrogenic phenotype, supporting contractile phenotype via microRNAs (e.g., miR-145, miR-145-5p, miR-92a), and inhibiting VSMC proliferation, migration, and apoptosis; 4) enhance the action of protective microRNAs that maintain VSMC contractile phenotype (miR-145, miR-145-5p), protect against oxidative stress and vascular remodeling (miR-26a), inhibit NF-κB pathway (miR-424-5p), suppress Toll-like receptor signaling and inflammation (miR-146a), and counteract endothelial activation (inhibition of miR-92a); 5) inhibit histone methyltransferases such as SET7/9 that activate pro-inflammatory genes (NF-κB, MCP-1, TNFα, ICAM-1); 6) suppress expression of genes associated with NADPH oxidase and NLRP3 inflammasome; 7) regulate expression of long non-coding RNAs such as ANRIL; 8) modulate autophagy and immune cell responses by reducing M1 macrophage activity and pro-inflammatory phenotype through regulation of AMPK/mTOR/ULK1 pathway involving miR-145-5p → CaMKIIδ, affecting macrophage autophagy via DNA methylation of GAL8 and inhibition of TRPV4; and 9) reduce mechanical stress and alleviate its effects caused by disturbed blood flow by stabilizing expression of shear stress-regulated microRNAs (e.g., TRPV4) such as miR-92a and miR-145 (Zaina et al., 2014; Zhuang et al., 2021).

Epigenetic changes occurring in platelets and PMPs, including within the gene encoding PAR-1 (i.e., the F2R gene), promote platelet activation. These changes may also occur at the level of DNA methylation, histone modifications, and microRNA activity, and they contribute to the progression of atherosclerosis (Patsouras and Vlachoyiannopoulos, 2019). Epigenetic changes in the methylation of the PAR-1 gene likely occur through the same mechanisms as those in inflammatory genes such as MCP-1. Histone modifications can influence PAR-1 expression, while microRNAs like miR-126 and miR-92a have been identified as regulators of PAR-1 expression (Sunderland et al., 2017; Demine et al., 2018). Furthermore, it is likely that the function of PAR-1 may be influenced by epigenetic regulators that can link DNA methylation with histone modifications, such as MeCP2 (methyl-CpG-binding protein 2), which increases DNA methylation and modifies histones like H3K9me2. Although not yet conclusively proven, epigenetic modifications within the PAR-1 gene–such as DNA methylation and interactions with microRNAs–may be associated with polymorphisms of this gene. These mechanisms may influence the expression of PAR-1, which is significant in the context of atherosclerosis and other cardiovascular diseases, and understanding these interactions will enable the development of new therapeutic strategies. DNA methylation within the PAR-1 gene may potentially lead to the occurrence of the rs2227744G>A polymorphism in the PAR-1 promoter region, where the presence of the minor allele (A) increases PAR-1 gene expression by 2.6-fold compared to G/G homozygotes. Moreover, the rs1801719 C/T polymorphism located in the 3′untranslated region (3′UTR) of PAR-1 influences microRNA binding sites, potentially regulating PAR-1 gene expression and modulating interactions with microRNAs, which may have important implications in the context of atherosclerosis (Zhou et al., 2009; Alkozi et al., 2017; Cañadas-Garre et al., 2019).

Polymorphisms of genes encoding platelet receptors, including PAR-1, may also be caused by changes other than just epigenetic modifications. Most of these genes have already been sequenced, enabling the identification of polymorphisms in their coding and regulatory regions. The consequences of these polymorphisms for platelet function and their role in predispositions to excessive bleeding or thrombosis have also been discovered–thrombin activates both endothelial cells and platelets via PAR-1, and the density of this receptor on the cell surface can have significant implications for hemostasis or the risk of excessive bleeding. he PAR-1 gene is approximately 27 kb in length and consists of 2 exons separated by a large intron (∼22 kb). It is located on chromosome 5q11.2-q13.3 (Chromosomal assignment of the human thrombin receptor gene: localization to region q13 of chromosome 5) (Vassallo et al., 1992; Kahn et al., 1998). The first exon encodes the signal peptide sequence and the N-terminal sequence of the receptor, while the second, larger exon encodes the remainder of the receptor (Kahn et al., 1998). Studies of the regulatory region sequence did not reveal the presence of characteristic TATA and CAAT sequences, which are often typical for genes encoding G protein-coupled receptors (De Backer et al., 1998). However, many potential regulatory motifs were found, such as SP1, Ets, TEF-1 (transcription enhancer factor-1), and GATA. Functional analysis of the promoter demonstrated that two sequences, SP-1 and AP-1 (activator protein 1), play a significant role in its basal activity (Wu et al., 1998). The analysis of genetic variations in the PAR-1 gene and their role in regulating both basal and induced transcription may have a significant impact on the risk of thrombosis. As part of the large-scale clinical PATHROS study (Paris Thrombosis Study), researchers investigated polymorphisms in the regulatory regions of the PAR-1 gene, including its promoter and the exon–intron boundary region, which may influence the gene’s expression (Arnaud et al., 2000). Two polymorphisms have been identified in the 5′regulatory region. The first is a C-to-T substitution located 1,426 base pairs upstream of the transcription start site (−1426 C/T). The second polymorphism is a 13-base pair insertion before the sequence at position −506: 5′-CGGCCGCGGGAAG-3′ (−506 I/D, where I denotes insertion and D denotes deletion) (Arnaud et al., 2000; Dupont et al., 2003). A polymorphism may also occur within the intronic sequence (IVS), where an A-to-T transversion has been identified, located 14 nucleotides upstream of the start of exon 2 (IVSn-14 A/T) (Arnaud et al., 2000).

## 2 Platelets (PLTs) and platelet-derived microparticles (PMPs)

**FIGURE 1:**
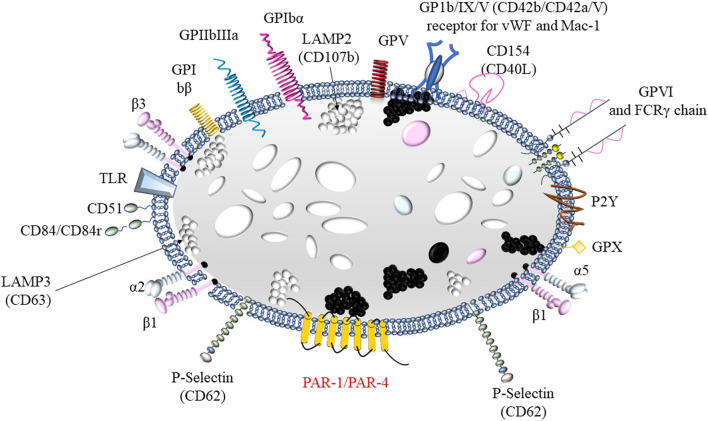
Structure of a platelet including membrane glycoproteins: 1) fibrinogen receptor: GPIa, GPIIb/IIIa (αIIbβ3) or VLA-5; 2) receptor for von Willebrand factor and Mac-1GPIb/IX/V; 3) laminin receptor: GPIc’-IIa or VLA-6 and 4) collagen receptors α2β1 and GPVI.

Platelets (PLTs), as cellular elements primarily involved in the formation of the primary hemostatic plug, play a crucial role in hemostasis. Due to the presence of adhesive glycoproteins (GPs) in their cell membranes, which act as receptors, PLTs are capable of binding to various molecules. For example, glycoprotein Ia (GPIa) binds to collagen–the main component of the subendothelial layer. Glycoprotein Ib (GPIb) and the glycoprotein IIb/IIIa complex (GPIIb/IIIa) are involved in the binding of PLTs to von Willebrand factor (vWF), which is secreted from the granules of both PLTs and endothelial cells and is essential for the proper adhesion of PLTs to collagen (Figure 1). Additionally, GPIIb/IIIa also serves as a receptor for fibrinogen, a single molecule of which binds two PLTs during the aggregation process (Mann et al., 2006). Glycoproteins serve as receptors for numerous factors that either activate or inhibit platelet (PLT) function (Zapata et al., 2014).

The platelet (PLT) cell membrane forms an open canalicular system directed toward the cytoplasm, through which the contents of intracellular granules are released. These granules include α-granules, dense granules, lysosomes, and peroxisomes. The most abundant are the α-granules, which contain platelet factor 4 (PF4), platelet-derived growth factor (PDGF), β-thrombomodulin, fibrinogen, von Willebrand factor (vWF), coagulation factor V, high-molecular-weight kininogen, fibronectin, and α1-antitrypsin. Dense granules store ADP, ATP, serotonin, catecholamines, as well as calcium and magnesium ions (Zawilska K, 2019; George JN, 2000). The contents of α- and δ-granules are released into the surrounding environment as a result of platelet (PLT) activation, which occurs after their adhesion to the subendothelial matrix. Activated PLTs express P-selectin on their surface and expose negatively charged phospholipids, which bind coagulation factors (George JN, 2000). Within the cytoplasm, directly beneath the cell membrane, there are fibrillar proteins with contractile properties, such as myosin, actin, and α- and β-tubulin. These proteins form the platelet cytoskeleton, which maintains the discoid shape of resting platelets and enables shape change during activation (George JN, 2000). This shape change involves the translocation of glycoproteins between the outer and inner membranes.

The primary function of platelets (PLTs) is to form a mechanical plug that prevents the free outflow of blood from a damaged blood vessel. In vessels where blood flow generates significant shear stress (high shear), such as in large arteries, platelets bind to the subendothelial matrix vWF–this interaction is mediated by the Ib/IX/V complex. In vessels with slower blood flow, platelets bind directly to collagen through their GP Ia/IIa receptor. Stronger attachment of platelets to the injured vessel wall occurs through additional interactions: GPIIb/IIIa with vWF, and GPVI and integrin α1/β2 with collagen and other components of the subendothelial matrix. As a result of these interactions, platelets become activated. Activated PLTs undergo morphological changes, becoming irregularly shaped cells with numerous protrusions. Simultaneously, substances stored in the intracellular granules are released through the open canalicular system. One of the effects of platelet activation is a conformational change of GPIIb/IIIa, the fibrinogen receptor, which in its dimeric form allows the binding of two platelets together.

Platelets (PLTs) activation is a complex process involving various factors from the coagulation and fibrinolysis systems. As a result of endothelial damage, PLTs begin to adhere to adhesive proteins such as collagen, fibronectin, and laminin (Creager Mark, 2008) (the first stage of primary hemostasis) (Shibata et al., 2008), thus providing protection against excessive blood loss. Additionally, there is a local narrowing of blood flow, which facilitates PLTs activation and limits blood loss (Michiels, 2003). The endothelium produces factors involved in coagulation and fibrinolysis processes, such as tissue factor (TF), which initiates blood coagulation. However, properly functioning endothelial cells also exhibit strong anticoagulant properties, maintaining blood fluidity within the circulation. Endothelial cells are also the site of production for activators and inhibitors of plasminogen, vWF, and factors regulating vascular tone and influencing PLTs adhesion, activation, and aggregation, such as prostacyclin, nitric oxide (NO), endothelin, and platelet-activating factor (PAF). Prostacyclin and NO cause vasodilation by relaxing smooth muscles, leading to a decrease in blood pressure. Endothelin, on the other hand, exerts vasoconstrictive effects. Prostacyclin is a potent inhibitor of platelet activation and aggregation, while NO inhibits platelet adhesion (Ismaeel et al., 2020). Endothelin has no effect on PLTs activity. Endothelial cells produce and present thrombomodulin (TM) and the endothelial protein C receptor (EPCR) on their surface, which contribute to the anticoagulant protein C system. The endothelial surface also contains ectonucleotidases that break down ADP into adenosine, which inhibits PLTs aggregation.

PLTs and coagulation mechanisms are key factors influencing the development of atherosclerosis and the formation of clots associated with this disease (Gawaz et al., 2005). Microtears and the subsequent thrombosis play a significant role in the destabilization of atherosclerotic plaques, leading to plaque growth and destabilization (Finn et al., 2010). The modulation of the atherosclerotic process involves both hemostatic and non-hemostatic (pro-inflammatory) actions of proteins from the hemostatic system. In the early stages of inflammatory changes, local thrombin or fibrin formation may be linked to the body’s natural defense mechanism. Later, a multifaceted role of hemostasis can be observed in the initiation and progression of atherosclerotic plaque development. Proteins from this system play a strategic role in maintaining or reducing the stability of the plaque, ultimately leading to thrombotic complications.

PLTs play a significant role in promoting processes associated with atherosclerosis and act as a link between hemostasis and the inflammatory state of atherosclerosis (Gawaz et al., 2005). The main mechanism of platelet adhesion to damaged vascular endothelium is the binding of von Willebrand factor (vWF), secreted from both PLTs and the endothelium, to its receptor and the GPI on PLTs. Stable adhesion is achieved through integrin β3. After binding to the vessel wall surface, platelets release pro-atherogenic mediators, such as cytokines, chemokines, growth factors, adhesive molecules, and coagulation factors. Increased expression of P-selectin on both PLTs and endothelial cell surfaces leads to enhanced interactions with P-selectin glycoprotein ligand 1, present on the surface of circulating leukocytes (LEU). The interaction between PLTs and LEUs (monocytes or neutrophils), dendritic cells, and progenitor cells leads to the formation of cellular aggregates. These aggregates support further activation, adhesion, and transmigration of leukocytes, driving the development and progression of atherosclerotic plaques (Ferrari and Fox, 2009).

Extracellular vesicles are small vesicles released by cells into the extracellular environment (Boilard et al., 2015). Extracellular vesicles stored in multivesicular bodies (or α-granules in platelets) and released via exocytosis range in size from 50 to 150 nm and are called exosomes. Extracellular vesicles formed by budding and shedding from the plasma membrane are larger (100–1000 nm) and are referred to as microvesicles (MPs). Extracellular vesicles released from apoptotic cells are known as apoptotic bodies or apoptotic vesicles (Boilard et al., 2015). MPs are membrane vesicles with a diameter ranging from 0.1 to 1.0 µm, secreted by platelets, erythrocytes, monocytes, lymphocytes, and endothelial cells. MPs are characterized by the lack of a cell nucleus and an inability to synthesize their own proteins, but they can contain a variety of biologically active substances such as enzymes, membrane proteins, genetic material, adhesion proteins, coagulation factors, and membrane lipids. The origin of microparticles has been determined based on the expression of surface antigens (Clusters of Differentiation, CD) characteristic of the cells from which they were derived: erythrocyte-derived MPs–CD235+ (ErMPs); platelet-derived MPs–CD42a+ (PMPs); leukocyte-derived MPs–CD45^+^ (LMPs); and endothelial cell-derived MPs–CD144+(EMPs) (Barteneva et al., 2013). The process of microparticles release from eukaryotic cells is a natural, physiological phenomenon that occurs during cell maturation and aging (Piccin et al., 2007). MPs regulate and coordinate many processes: inflammation, neovascularization, coagulation, and fibrinolysis. They also link coagulation with local inflammation (Morel et al., 2004). Furthermore, they play an important role in the occurrence of cancer. PMPs are biomarkers of platelet activation and the intensification of the inflammatory process. MPs are similar to exosomes, which are also secreted by various cells. The differences between these two types of vesicles are presented in Table 1.

**TABLE 1:** Comparison of exosomes and MPs (Zawilska K., 2019).

Feature	Exosomes	MPs
Size	30–150 nm	100 nm – 1 µm
origin	Produced by multivesicular bodies (MVBs) of the endosomal system	Bud from the plasma membrane during activation or apoptosis
lipid composition	Cholesterol Sphingomyelin Ceramides Phosphatidylserine (PS) Lipid rafts	Sphingomyelin Phosphatidylserine (PS) Phosphatidic acid (PA) Arachidonic acid
proteins	Proteins of DNA, proteins of mRNA Proteins of microRNA Integrin tetraspanin family proteins (CD9, CD81, D63), heat shock proteins (HSPs) GTPases, annexin, flotillin	Cell adhesion proteins (VE-cadherin, E-selectin, sialomucin), proteins of DNA, Proteins of mRNA Proteins of miRNA
Others membrane markers	Alix TSG101	CD42a CD41, annexin V
Diagnostic use	In various diseases	Cardiovascular risk

PMPs are large lipid–protein complexes abundantly present in the blood, secreted by activated platelets (PLTs) or megakaryocytes (Dean et al., 2009). PMPs originating from megakaryocytes, in contrast to those released from activated PLTs, contain CD62P, LAMP-1, and immunoreceptor-based activation motif (ITAM)-containing receptors (Flaumenhaft et al., 2009). PMPs are heterogeneous in size, protein and lipid content, and functional potential (e.g., distinct cytokine and miRNA profiles). Based on protein composition, protein-to-lipid ratio, and functional effects on platelets and endothelial cells, they have been divided into four size classes (Dean et al., 2009). The heterogeneity of PMPs depends on the type of activating stimulus for PLTs, which is important in the context of targeted therapies and disease biomarkers (Boilard et al., 2015). PMPs are generated during platelet activation or apoptosis, and their release is triggered by stimuli such as contact with collagen, thrombin, ADP, or PAF (platelet-activating factor), oxidative stress, high protease activity, mechanical shear stress (e.g., in pulmonary circulation or with artificial valves), infections, autoimmune diseases, and cancer. These interactions lead to cytoskeletal rearrangement and the translocation of phosphatidylserine (PS) from the inner to the outer leaflet of the plasma membrane, ultimately resulting in membrane budding and the formation of microparticles (Varon and Shai, 2015). Like other MPs, PMPs range in size from 100 to 1000 nm, classifying them as microvesicles, not exosomes. PMPs are surrounded by a lipid bilayer and contain platelet-specific membrane proteins such as CD41 (GPIIb), CD42b (GPIbα), P-selectin (CD62P), coagulation factors (Va, VIII), chemokines, cytokines (e.g., RANTES, PF4), integrins, selectins, microRNAs and mRNAs (allowing modulation of target cell function), as well as phosphatidylserine (PS), which provides a surface for coagulation complexes, and enzymes like metalloproteinase activators. PMPs promote the adhesion of platelets and leukocytes to the subendothelial matrix. They can also interact with leukocytes, endothelial cells, monocytes, and cancer cells, influencing their pro-inflammatory, pro-thrombotic, and immunomodulatory responses. PMPs are highly procoagulant, due to the exposure of phosphatidylserine and transfer of coagulation factors (PMPs are more procoagulant than intact platelets), and pro-inflammatory, as they promote leukocyte and endothelial cell activation and cytokine release (Gidaro et al., 2023). Additionally, PMPs stimulate monocyte migration and activation, cause endothelial damage (atherogenic effect), and support angiogenesis and tumor metastasis (carcinogenic effect) (Varon and Shai, 2015; Kassassir et al., 2023). They also enhance immune responses and may serve as a source of autoantigens (autoimmune activity) (Rother et al., 2022). Therefore, PMPs are potential biomarkers of inflammation, cardiovascular diseases, and cancers. Their levels are elevated in conditions such as stroke, myocardial infarction, disseminated intravascular coagulation (DIC), lupus, and antiphospholipid syndrome (Kassassir et al., 2023). They also represent a potential therapeutic target in thrombotic disorders (Kailashiya et al., 2019).

PMPs are formed during the physiological process of coagulation. They account for 70%–90% of all PMPs present in the blood (Siljander, 2011) and contain a high concentration of phospholipids, which are essential for the coagulation process. Similar to PLTs, PMPs display binding sites for collagen and vWF on their surface, allowing them to adhere to fibrin and thereby accelerating clot formation (Owens and MacKman, 2011). Under procoagulant conditions or during chronic inflammatory states, an increased release of PMP is observed from activated or apoptotic cells (Maślanka K, 2010). Prior to membrane remodeling and the release of PMP, PLTs undergo an intracellular increase in Ca^2+^ concentration. This response is triggered by elevated shear stress, the presence of reactive oxygen species, adenosine diphosphate (ADP) released by activated PLTs, and the expression of CD40 ligand on the surface of activated T lymphocytes (Morel et al., 2008). As a result, calcium-dependent enzymes are activated, disrupting the asymmetric distribution of phospholipids in the cell membrane, which leads to the translocation of phosphatidylserine from the inner to the outer leaflet of the membrane (Morel et al., 2008). At the same time, cytoskeletal destabilization occurs, enabling the formation and shedding of microparticle vesicles from the cell membrane (Morel et al., 2008). The process begins with the stimulation of PLTs through ligand binding to a receptor or activation of a calcium channel. The receptor transmits a signal inward and/or triggers calcium influx. Elevated calcium levels activate calpain, which inhibits F-actin and releases spectrin from submembrane compartments, or induces RhoA phosphorylation. The asymmetry of membrane lipids is regulated by the cooperative actions of three transporters: an ATP-dependent aminophospholipid-specific flippase that rapidly translocates PS and PE from the outer to the inner leaflet of the membrane; an ATP-dependent nonspecific lipid floppase that slowly moves lipids from the inner to the outer leaflet; and a Ca^2+^-dependent nonspecific lipid scramblase that allows for the random movement of lipids between both leaflets. In the final stage, contractions of myosin II occur (Konkolewska et al., 2014).

Aminophospholipids present on the surface of PMPs and endothelial microparticles (EMPs) contain numerous binding sites for coagulation factors IXa, VIII, Va, and IIa. Consequently, the activation of blood coagulation proteins can occur not only on intact platelets but also on microparticles (Berckmans et al., 2002). Factor Va, as part of the complex with factor Xa, forms the prothrombinase complex, which, in the presence of calcium ions, participates in the conversion of prothrombin to thrombin–a key enzyme in the coagulation process that transforms fibrinogen into fibrin (Łopaciuk S, 2003). Similarly, vWF associated with EMPs binds more readily to vWF receptors on platelets than free vWF does (Jy et al., 2005). The procoagulant activity of PMPs is influenced by the presence of P-selectin and tissue factor (TF) on their surface, and by the presence of P-selectin glycoprotein ligand-1 (PSGL-1) on microparticles derived from monocytes (Piccin et al., 2007). Under physiological conditions, circulating microparticles also serve an anticoagulant function. Microparticles exposing phosphatidylserine on their surface participate in thrombin formation, which subsequently activates protein C–a key player in the degradation of coagulation factors Va and VIIIa.

PMPs more frequently bind to granulocytes than to lymphocytes, and in both cases, they induce increased expression of the adhesion molecule CD11b as well as enhanced phagocytic activity. Inflammatory markers, traditionally considered soluble molecules, have also been found to be associated with microparticles, such as Platelet Endothelial Cell Adhesion Molecule-1 (CD31, PECAM-1). Studies involving microparticles derived from human umbilical vein endothelial cells (HUVECs) have demonstrated increased expression of the adhesion molecule ICAM-1 (Barry et al., 1998). *In vitro* studies have shown that endothelial microparticles (EMPs), after stimulation with TNF-α, bind to human monocytes via ICAM-1 and also stimulate the production of tissue factor (TF) (Sabatier et al., 2002). Microparticles released from multinucleated cells can induce the release of pro-inflammatory cytokines from endothelial cells (Mesri and Altieri, 1999). During apoptosis, a significant increase in intracellular Ca^2+^ concentration and the activity of proteolytic enzymes leads to the degradation of DNA and proteins, as well as the release of membrane microparticles (Piccin et al., 2007).

Both platelets PLTs and PMPs can be activated by various factors through different molecular mechanisms. PLTs/PMPs activation is a complex process influenced by various biochemical and mechanical signals (Table 2). Coagulation factors, such as thrombin, activate platelets by binding to protease-activated receptors (PAR-1 and PAR-4), inducing shape change, granule release, and aggregation. In response to vascular injury, exposed collagen binds to GPVI and integrin α2β1 receptors, initiating adhesion and activation, while von Willebrand Factor (vWF) facilitates adhesion through the GPIb-IX-V complex. Platelet-derived signals play a key role in amplification. ADP acts on P2Y1 and P2Y12 receptors, promoting aggregation and recruitment of additional platelets. Thromboxane A_2_ (TXA_2_), synthesized via the COX-1 pathway, further enhances activation and causes vasoconstriction. Neurohormonal mediators, such as epinephrine, stimulate α2A-adrenergic receptors, intensifying the response. Inflammatory mediators like cytokines (e.g., IL-1β, TNF-α) increase platelet reactivity and interaction with immune cells, while chemokines (e.g., MCP-1) facilitate platelet-leukocyte interactions. Additionally, mechanical or chemical stress, such as shear forces and oxidative stress, can trigger platelet activation through receptor-independent mechanisms. Finally, genetic and epigenetic factors, including microRNAs (e.g., miR-126, miR-223), DNA methylation, and histone modifications, regulate the expression of genes involved in platelet function, highlighting the multilayered control of platelet activation.

**TABLE 2:** Factors influencing PLTs and PMPs activation.

Molecular level	Factor	Mechanism of PLTs/PMPs action
Coagulation Factors	Thrombin	Activates PAR-1 and PAR-4 receptors; induces shape change, granule release, aggregation
Vascular Injury Signals	Collagen	Binds to GPVI and integrin α2β1 receptors; initiates platelet adhesion and activation
	Von Willebrand Factor (vWF)	Mediates adhesion via GPIb-IX-V complex at sites of endothelial damage
Platelet-Derived Signals	ADP (Adenosine Diphosphate)	Acts on P2Y1 and P2Y12 receptors; promotes aggregation and recruitment of more platelets
	Thromboxane A_2_ (TXA_2_)	Produced via COX-1 pathway; enhances platelet activation and vasoconstriction
Neurohormonal	Epinephrine (Adrenaline)	Stimulates α2A-adrenergic receptors; potentiates platelet activation
Inflammatory Mediators	Cytokines (e.g., IL-1β, TNF-α)	Enhance platelet reactivity and interaction with immune cells
	Chemokines (e.g., MCP-1)	Promote platelet-leukocyte interactions
Mechanical/Chemical Stress	Shear stress, toxins, oxidative stress	Induce platelet activation via receptor-independent mechanisms
Genetic/Epigenetic	microRNAs (e.g., miR-126, miR-223)	Modulate gene expression related to platelet function
	DNA methylation, histone modification	Influence expression of platelet activation-related genes

## 3 Protease-activated receptors (PARs)

One of the mechanisms of PLTs and PMPs activation involves the activation of protease-activated receptors (PARs) belonging to G protein-coupled receptors (GPCRs) constitute the largest superfamily of cell surface receptors and are crucial for signal transduction within the body (Fredriksson et al., 2003). They usually consist of 415 amino acids and are single polypeptide chains containing seven hydrophobic transmembrane domains, five of which are functional: the N-terminal extracellular domain, the transmembrane domain composed of seven α-helices, the intracellular domain, and the C-terminal domain, which is cytoplasmic and facilitates G protein binding (Rasmussen et al., 1991). Activation of PAR receptors can occur through various mechanisms, leading to opposing effects–either pro-inflammatory or anti-inflammatory (Tokmakova et al., 2022). Activation of protease-activated receptors (PARs) can proceed via different mechanisms, resulting in contrasting cellular outcomes–ranging from pro-inflammatory to anti-inflammatory effects. These divergent responses depend on the type of protease involved, the specific cleavage site on the receptor, and the downstream signaling pathway that becomes activated. Usually, activation of PAR receptors carries out by classical (canonical) activation of PARs initiates intracellular signaling pathways, which may proceed through different routes depending on the type of G protein involved in the process. This variability in cellular response to thrombin results from the diversity of signaling pathways (Ossovskaya and Bunnett, 2004). Activated PARs initiate processes leading to the release of inflammatory mediators, leukocyte recruitment, and the formation of edema. Thrombin (or other protease) is not a ligand for PAR, but a protease that, by cleaving the extracellular N-terminal domain of the receptor, leads to the formation of a new N-terminus. The new N-terminal domain binds to the central loop of the extracellular domain of the same receptor, resulting in receptor activation (Trejo, 2003). The released oligopeptide may have some physiological role, but there is no definitive evidence for this. Moreover, PARs are activated through proteolytic cleavage by various proteases, which exposes a “tethered ligand” that initiates intracellular signaling. This occurs according to the concept of biased agonism, whereby different proteases or ligands can elicit distinct cellular responses–ranging from pro-inflammatory and destructive to cytoprotective and anti-inflammatory–depending on the signaling context (Tokmakova et al., 2022). Depending on the specific agonist, the receptor may preferentially activate one of several signaling pathways–for example, G protein–mediated (pro-inflammatory) or β-arrestin–mediated (anti-inflammatory). Following GPCR phosphorylation by GRKs, β-arrestin binds to the receptor, thereby inhibiting further G protein signaling and leading to internalization. In addition, β-arrestin also acts as a scaffold protein, initiating independent signaling cascades such as MAPK/ERK activation (Lymperopoulos et al., 2012), and promotes barrier stabilization, cytoprotective activity, and reduced permeability, particularly in response to activated protein C (APC) (Griffin et al., 2016).

So far, four types of PARs have been identified: PAR-1, PAR-2, PAR-3, and PAR-4, which share approximately 30% sequence homology. Differences exist in the N-terminal (extracellular) sequence, which contains the protease-binding and peptide bond cleavage site, determining which protease is capable of activating a given receptor. Thrombin is able to activate PAR-1, PAR-3, and PAR-4, while the primary activator of PAR-2 is trypsin (Ossovskaya and Bunnett, 2004). PARs can be activated by different proteases, depending on the environment and the presence of cofactors (Han and Nieman, 2020). The proteases that activate specific PAR receptors are presented in Table 3.

**TABLE 3:** Protease specificity for PAR receptors (Ossovskaya and Bunnett, 2004).

Enzyme	PAR-1	PAR-2	PAR-3	PAR-4
Activating Protease	Thrombin	Tryptase	Thrombin	Thrombin
	Factor XA	Trypsin		Trypsin
	Activated protein C (APC)	Kalikrein 14		Kalikrein 14
	Granzyme A	Factor Xa		Katepsin G
	Gingipain R	Gingipain R		Gingipain R
	Trypsin	MT-SP1		
	Matrix Metalloproteinase (MMP)	Proteinase-3 Factor VIIa		

**FIGURE 2:**
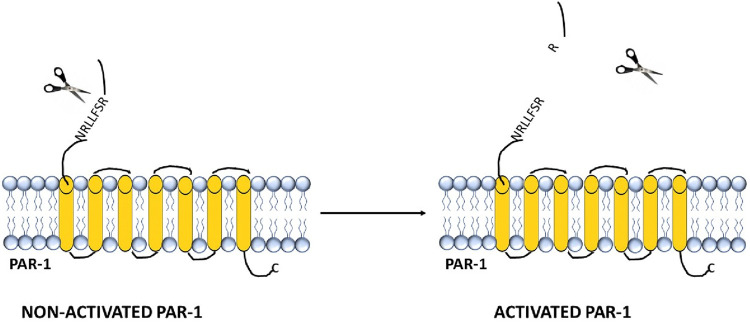
Structure and activation of the thrombin receptor PAR-1.

The detailed mechanism of PAR activation has been well elucidated for the interaction between PAR-1 and thrombin (Figure 2).

The binding of this enzyme leads to the formation of a negatively charged part of the receptor molecule (homologous to the hirudin domain) and a conserved area within the α-thrombin molecule known as the Anion-Binding Site. In PAR-1, upon activation, the binding between Arg41 and Ser42 in the sequence TLDPRSFLLR is disrupted. The resulting exposed sequence becomes a “tethered” ligand, which intracellularly binds to the residue 42SFLLRN47 in the conserved region of the receptor’s second loop, leading to the activation of transmembrane cell signaling (Hakanishi-Matsui et al., 2000).

PAR-1 plays a key role in the thrombin signaling pathway (Rasmussen et al., 1991; Vu et al., 1991). Thrombin-activated PAR-1 is expressed on the surface of a variety of cell types, primarily those associated with the vascular wall–endothelial cells, fibroblasts, and smooth muscle cells–as well as on all types of blood cells, including platelets, neutrophils, and macrophages (Austin et al., 2013). It is also found in epithelial cells, neurons, astrocytes, cells of the immune system (Malito et al., 2013), and in mast cells and macrophages within the tumor microenvironment (Austin et al., 2013; Aisiku et al., 2015). PAR-1 exhibits moderate affinity for thrombin (Kd ∼10 nM) and is present on the cell surface in approximately 1,500 to 2,000 copies per cell (Macfarlane et al., 2001). The thrombin interaction site on the PAR-1 receptor is located within a hirudin-like domain. The extracellular N-terminal region of PAR-1 includes potential sites for phosphorylation, palmitoylation, and N-glycosylation, as well as a critical cleavage site for thrombin. Upon cleavage of PAR-1 at Arginine 41 and Serine 42, a hidden ligand is exposed, beginning with the sequence SFLLRN, which starts at Serine 42 – the cleavage site recognized by enzymes like thrombin (Polpili and AuthorAnonymous, 2019).

**FIGURE 3:**
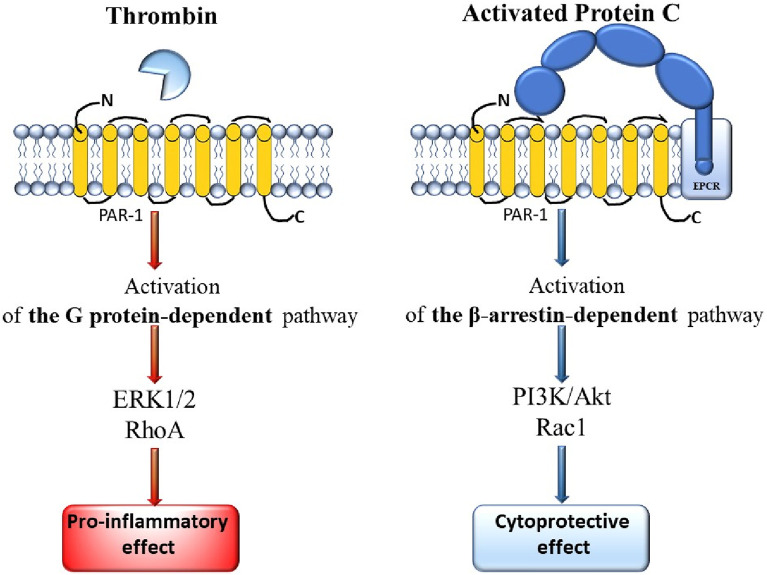
Comparison of the mechanisms of PAR-1 activation by thrombin and by APC.

PAR-1 has a longer N-terminus compared to PAR-4 (81 amino acids versus 65 amino acids). Thrombin cleavage shortens the PAR-1 domain by 20 amino acids, exposing the ligand sequence SFLLRN. PAR-1 contains a sequence similar to hirudin, which interacts with the exosite I of thrombin and inhibits the enzyme in its active conformation. As a result, PAR-1 is a substrate that can be activated by subnanomolar concentrations of thrombin (Han and Nieman, 2020). The binding of thrombin to the receptor is irreversible and always occurs in a 1:1 ratio, which means that the degree of cell activation depends on the local concentration of thrombin (Ossovskaya and Bunnett, 2004). Activation of PAR-1 can also be initiated by activated protein C (APC), where activation by thrombin results in an inflammatory state, while interaction with APC triggers cytoprotective mechanisms (which occur with lower catalytic efficiency than those involving thrombin) (Ludeman et al., 2005). APC cleaves PAR-1 either at Arg41 or at Arg46 (preferred) (Malaquin et al., 2013). A comparison of PAR-1 activation by thrombin and by APC is presented in Figure 3.

Thrombin cleaves PAR-1 at the R41-S42 site, which primarily initiates G protein signaling pathways, leading to pro-inflammatory signaling, disruption of barriers, and increased cell permeability. APC bound to thrombomodulin (TM) and associated with endothelial protein C receptor (EPCR), cleaves PAR-1 at the R46-N47 site. The bound ligand (generated in the presence of APC) initiates β-arrestin pathways and an anti-inflammatory response, as well as disrupts endothelial barriers (increased permeability) (Han and Nieman, 2020). Under physiological conditions, the endothelial protein C receptor (EPCR) plays a crucial role as a cofactor facilitating the cleavage of PAR-1 by APC (Riewald et al., 2002). Thrombin, protein C, thrombomodulin, and GPCRs co-localize with PAR-1 on the surface of endothelial cells (Russo et al., 2009).

The process of PAR-1 activation can also be initiated by matrix metalloproteinases (MMP-1), which occurs through a different mechanism than that involving thrombin. PAR-1 is cleaved at the D39-P40 site, generating a ligand with a chain length that is 2 amino acids longer than that produced by thrombin (PR-SFLLRN). The activated ligand activates G12/13 proteins, Rho-GTP, leads to p38 phosphorylation, and triggers the MAPK signaling pathway. This process alters the shape and motility of platelets, although to a lesser extent than the activation of PAR-1 via thrombin (Austin et al., 2013).

PAR-2 (a G protein-coupled receptor (GPCR), similar to PAR-1), contains seven transmembrane domains, including an N-terminal domain that serves as the recognition site for proteases. It also includes three extracellular loops, three intracellular loops, and an intracellular COOH terminus (Jin et al., 2016). PAR-2 is primarily found on endothelial cells, fibroblasts, keratinocytes, epithelial cells, monocytes, neutrophils, neurons, and glial cells (Xu et al., 2024). It is a versatile transmembrane receptor that detects and responds to active proteases present in the cellular microenvironment. It plays an essential role in maintaining homeostasis as well as in diseases such as asthma, lung injury, inflammatory bowel diseases, irritable bowel syndrome, neurogenic inflammation, and cancer (Pawar et al., 2019). In contrast to other PARs, its main activator is trypsin, not thrombin. Trypsin usually cleaves PAR-2 between Arg36 and Ser37. This cleavage exposes a new N-terminus that begins with the sequence SLIGKV, which acts as a “tethered ligand” that binds to the second extracellular docking loop (ECL2) of the receptor, induces a conformational change, and initiates intracellular signaling—this is referred to as canonical activation (Rayees et al., 2019). There is also a non-canonical activation pathway of PAR-2, mediated by certain proteases such as neutrophil elastase (Ser67/Val68) (Zhao et al., 2019), chymase (Gly35/Arg36) (Hollenberg et al., 2014), or mast cell protease 3, which can activate PAR-2 either without cleaving it at the canonical site or by partially activating the receptor. This results in different (often milder or cytoprotective) cellular responses. Likewise, proteases such as Der p3 and Der p9 (from house dust mites), Per a7 (from cockroach allergens), and KLK5, KLK14 can proteolytically activate PAR-2, disrupting epidermal barrier homeostasis, innate and adaptive immunity, leukocyte recruitment, pigmentation, tumor development, and cutaneous paresthesia (Sastre et al., 2025). PAR-2 activation triggers various signal transduction cascades involving: Gq protein (activation of phospholipase C and increase in intracellular Ca^2+^), Gi protein (inhibition of adenylate cyclase), MAPK pathway (ERK1/2), initiating the expression of pro-inflammatory genes. These signaling pathways can involve interactions with receptor tyrosine kinases (e.g., epidermal growth factor receptor–EGFR, platelet-derived growth factor receptors–PDGFR, vascular endothelial growth factor receptor–VEGFR) (Kawao et al., 2005), as well as TRP ion channels (e.g., transient receptor potential vanilloid 1 (TRPV1), TRPV4 (Grace et al., 2014), and transient receptor potential ankyrin 1 (TRPA1)), or via alternative gene expression pathways (e.g., NF-kB, Toll-like receptor 4 (TLR4)) (Johnson et al., 2016). The interaction between PAR-2 and TRP ion channels results in sustained Ca^2+^ influx from both the extracellular space and the endoplasmic reticulum, leading to increased intracellular Ca^2+^ levels. Moreover, hundreds of genes regulated by PAR-2 signaling have been linked to cell metabolism, cell cycle, MAPK signaling, inflammatory cytokine expression, and anti-complement functions (Kumar et al., 2017).

PAR-3 also belongs to the group of G protein-coupled receptors (GPCRs). Its expression is found, among others, on platelets, endothelial cells, and cells of the nervous and immune systems. PAR-3 has a typical GPCR structure – 7 transmembrane domains, an N-terminal (extracellular) domain, extracellular and intracellular loops, and a C-terminal (cytoplasmic) end. PAR-3 is activated by proteolytic enzymes (mainly thrombin), which cleave its extracellular domain by cutting the bond between Lys and Thr in the sequence TLPIKTFRGA, exposing a so-called “tethered ligand”—a peptide fragment that activates the receptor within the same molecule. In contrast, the activation of PAR-4 occurs through the cleavage of the bond between Arg and Gly in the sequence LPAPRGYGQV (Kawabata and Kuroda, 2000). Although thrombin is the main activator of PAR-3, unlike PAR-1, PAR-3 does not independently trigger a strong signaling response but acts as a co-receptor, primarily supporting the activation of PAR-4 (e.g., on platelets). Therefore, PAR-3 does not signal on its own but facilitates the transfer of thrombin to PAR-4, which then initiates the activation response (Sébert et al., 2019). PAR-3 binds thrombin via a hirudin-like domain, enabling the activation of PAR-4 even at low thrombin concentrations (Hakanishi-Matsui et al., 2000). However, individual studies have shown that PAR-3 is capable of autonomously signaling to induce IL-8 release, mediated by ERK1/2 phosphorylation, which actively contributes to inflammatory responses (Ostrowska and Reiser, 2008). Additionally, PAR-3 can be noncanonically activated by activated protein C (APC), leading to endothelial cell protection (Hamilton and Trejo, 2017).

The activation mechanism of PAR-4, similar to other receptors of the PAR family, involves proteolytic cleavage of the extracellular N-terminal domain of the receptor by specific proteolytic enzymes, primarily thrombin, leading to the exposure of the so-called “tethered ligand.” Cleavage occurs between Arg and Gly within the LPAPRGYGQV sequence (Kawabata and Kuroda, 2000), and the newly exposed GYPGQV sequence functions as a ligand that binds to the second extracellular loop (ECL2) of the same receptor. This conformational change in PAR-4 initiates an intracellular signaling cascade. Activated PAR-4 interacts with G proteins (especially Gq and G12/13), initiating: activation of phospholipase C (PLC), leading to increased intracellular calcium concentration [Ca^2+^], activation of the MAPK/ERK signaling pathway, activation of RhoA and the cytoskeleton, expression of pro-inflammatory genes, and platelet aggregation (Coughlin, 2005). It is important to note that, unlike PAR-1, PAR-4 lacks a hirudin-like domain–as a result, higher concentrations of thrombin are required to activate PAR-4. As previously mentioned, PAR-4 often functions in cooperation with PAR-3, which facilitates thrombin binding, especially on platelets. PAR-4 is cleaved exclusively at its canonical site by thrombin, trypsin, tissue kallikrein, plasmin, and cathepsin G, contributing to the facilitation of interactions between platelets (PLTs) and leukocytes (LEUs), including neutrophils (a subset of granulocytes) and monocytes (a subset of mononuclear cells) (Totani et al., 2010). Furthermore, PAR-4 has a shorter N-terminus than PAR-1 (65 vs. 81 amino acids), and its activation is slower but more sustained than that of PAR-1, which is relevant for platelet aggregation. It has also been shown that PAR-4 is involved in the activation of protein kinase C (PKC) substrates, which are essential for dense granule secretion in platelets. This granule release can be inhibited by PAR-4 antagonists. The contents of these granules have been linked to the pathogenesis of certain diseases. In addition, platelet-leukocyte (PLT-LEU) interactions and platelet P-selectin exposure following stimulation with PAR-4 agonists are also reduced upon inhibition of activated PAR-4 (Rigg et al., 2019). These findings indicate a PAR-4-specific promotion of platelet granule release and the formation of platelet-leukocyte aggregates. Therefore, pharmacological control of PAR-4 activity could potentially reduce pathological processes dependent on platelet granule secretion or PLT-LEU interactions (Rigg et al., 2019). Additionally, the PAR-4 inhibitor BMS-986120 has shown promising effects in animal models of thrombosis, as well as in a completed phase 2 clinical trial, in combination with aspirin, for the prevention of recurrent stroke (Wong et al., 2017).

### 3.1 Regulation of PARs

The signal transduction begins with the binding of the G protein to the activated PAR receptor in the cytoplasmic domain. PAR-1, PAR-2, and PAR-4 interact with both Gαi and Gαq subunits, leading to various effects within the cell. Gαq plays a major role as a ligand by activating the phospholipase C signaling pathway. This triggers the release of inositol 1,4,5-trisphosphate (IP3), which increases cytosolic Ca2+ concentration, and diacylglycerol (DAG), which activates protein kinase C (PKC) in the cell membrane. Calcium ions and PKC then activate several cellular pathways, including the Mitogen-Activated Protein Kinase (MAPK) pathway, which is regulated by calcium and protein kinase C. MAPK proteins, such as ERK1/2, control the activity of many substrates and play a crucial role in cell division and differentiation processes (Ramachandran et al., 2012). The Gαi subunit, in turn, acts as an inhibitor of adenylyl cyclase, leading to a reduction in cAMP synthesis. This causes the release of arachidonic acid by phospholipase A2 and, consequently, an increase in the concentration of prostaglandins E and F through activation of cyclooxygenase. The Gβγ subunits of G protein complexes, on the other hand, link PAR receptors to effector proteins such as potassium channels, non-receptor tyrosine kinases, and phosphatidylinositol 3-kinase, influencing cytoskeletal reorganization and cell motility (Martin et al., 2001).

The PAR-1 receptor is capable of interacting with both Gα12 and Gα13 subunits. Both of these subunits interact with guanine nucleotide exchange factors (GEFs), which activate Rho protein, thereby affecting cell morphology and migration. PARs bind thrombin irreversibly. Termination of PAR signaling occurs via the classical receptor desensitization mechanism. G protein-coupled receptor kinases (GRKs), primarily GRK2 and GRK3 — serine/threonine kinases—bind to the active PAR-1 receptor. These kinases phosphorylate the intracellular C-terminal tail of the receptor. As a result, β-arrestin translocates and binds to the phosphorylated tail, blocking the receptor’s interaction with G proteins and thus inhibiting signal transduction (Chromosomal assignment of the human thrombin receptor gene: localization to region q13 of chromosome 5 - PubMed, 2024; Ramachandran et al., 2012). β-arrestin binding prevents the formation of additional protein complexes between PAR and G proteins, leading to their rapid dissociation within a few seconds (Bourquard et al., 2015).

The released receptors are internalized within minutes through the action of β-arrestin and other adaptor proteins such as clathrin and adaptor protein 2 (AP2). The budding of the clathrin-coated vesicle from the cell membrane and its fusion with the early endosome is coordinated by the GTPase dynamin. Subsequently, the receptors are degraded lysosomally within the cell. Damaged receptors are replaced by new ones originating from the intracellular membrane system (Ossovskaya and Bunnett, 2004). In the absence of stimuli, PAR receptors continuously cycle between the cell membrane and the intracellular compartment, maintaining a stable pool of receptors on the cell surface.

Protease-activated receptors (PARs) undergo post-translational regulation primarily through phosphorylation, ubiquitination, and internalization (Frantz et al., 2012). These processes allow control over the timing, intensity, and nature of the cellular response to PAR activation. Receptor desensitization (G protein-mediated signaling), trafficking, and degradation enable cells to precisely modulate pro-inflammatory, pro-thrombotic, or cytoprotective signals, which helps maintain homeostasis and prevents chronic inflammation or thrombosis. Post-translational modifications thus ensure proper regulation of cellular signaling and prevent excessive or chronic activation. Phosphorylation of activated PARs by protein kinases known as G protein-coupled receptor kinases (GRKs) increases the affinity of PARs for β-arrestin proteins, enabling the termination of further G protein-mediated signaling (desensitization) (Karnam et al., 2021). This prevents excessive cellular activation and allows precise regulation of the cellular response. Phosphorylated PARs bound to β-arrestin can be ubiquitinated and subsequently targeted for degradation in lysosomes or proteasomes (Xing and Strange, 2010). This reduces the number of receptors available on the cell surface and allows for sustained signal silencing. Control of receptor numbers on the cell surface regulates the duration and intensity of signaling. In addition to ubiquitination, phosphorylated PARs bound to β-arrestin may be internalized (removed from the cell surface into the cell interior within endocytic vesicles) (Pedersen et al., 2021). This allows receptors to be separated from their ligands and G protein signaling partners, halting the signal. Internalization of PAR-1 and PAR-4 occurs via different pathways, influencing the duration and nature of the signal. Internalization can lead to receptor recycling back to the cell surface (allowing receptor reactivation) or degradation (resulting in permanent receptor loss). Thus, internalization enables modulation of the cellular response both in the short term (signal termination) and long term (regulation of receptor numbers).

Furthermore, platelet-derived microparticles (PMPs) act as precise regulators of PAR signaling, modulating its intensity, nature, and duration–an essential function in controlling inflammatory and hemostatic processes within blood vessels. PMPs influence the signaling pathways activated by PARs through several mechanisms. For example, they can selectively activate PARs via various proteases present on their surface, such as thrombin, cathepsin, or elastase (Taus et al., 2019). These proteases differentially activate PAR-1 or PAR-4 (Rajala and Griffin, 2025), potentially leading to divergent outcomes–ranging from pro-inflammatory to cytoprotective signaling. The type of protease and its cleavage site on the receptor determines the specific cellular response. Moreover, PMPs carry cytokines, chemokines, miRNAs, proteolytic enzymes, and growth factors, all of which can modulate PAR activity and downstream signaling. For instance, different miRNAs may suppress or enhance the expression of proteins involved in PAR-associated pathways, resulting in variable cellular responses (Xia et al., 2018). PMPs can also interact with various target cells–such as endothelial cells, leukocytes, or monocytes–altering how these cells respond to PAR signals. Depending on their molecular content and the inflammatory context, PMPs may either promote endothelial barrier stabilization or contribute to its disruption (Badimon et al., 2016). Additionally, PMPs may influence signal transduction by affecting the recruitment of adaptor proteins like β-arrestin, thereby determining whether PAR activation drives pro-inflammatory or anti-inflammatory cellular responses. Finally, PMPs can enhance or suppress several downstream pathways linked to PAR activation, including MAPK, NF-κB, and PI3K/Akt pathways, thereby impacting the balance between inflammation, regeneration, and thrombosis.

### 3.2 The role of PAR-1

PAR-1 has pro-inflammatory effects. Its interaction with thrombin *in vivo* induces the expression of surface molecules on endothelial cells, such as P-selectin and E-selectin, as well as adhesion proteins ICAM-1 and VCAM-1. It also leads to the production of pro-inflammatory cytokines, such as IL-6 and IL-8, platelet-activating factors (e.g., PAF), monocyte chemoattractant proteins (MCP-1), and activation of cyclooxygenase 2 (COX-2). These chemoattractant factors stimulate the migration of platelets and leukocytes to the site of their release. E-selectin, ICAM-1, and VCAM-1 facilitate the movement (“rolling”) of granulocytes, lymphocytes, macrophages, and platelets along the surface of endothelial cells. PAR-1 stimulation in monocytes, in turn, leads to increased release of pro-inflammatory cytokines (TNF-α, IL-1, IL-6, IL-8, and MCP-1), while simultaneously decreasing IL-12 secretion. Similarly, on T lymphocytes, activated PAR-1 promotes their proliferation and the production of pro-inflammatory cytokines.

The primary mechanism of the inflammatory response induced by thrombin is the activation of the nuclear transcription factor κB (Nuclear Factor Kappa-Light-Chain-Enhancer of Activated B cells, NF-κB) through the degradation of phosphorylated IκB via proteasomes. Deactivation of IκB leads to the expression of pro-inflammatory factors such as ICAM-1, VCAM-1, IL-1, IL-6, IL-8, TNFα, MCP-1, and PAF (Strukova, 2001). Additionally, thrombin stimulates the expression of decay-accelerating factor (DAF) in endothelial cells, which increases the rate of degradation of complement components C3 and C5 (Dugina et al., 2002). Moreover (as mentioned earlier), thrombin, through the activation of protein C, also exerts anti-inflammatory effects. Activated protein C (APC) forms a complex with endothelial protein C receptors (EPCR) on endothelial cells, leading to a reduction in the production of pro-inflammatory cytokines by these cells. APC also interacts with monocytes, inhibiting the increase of intracellular calcium levels and blocking the NF-κB pathway (Esmon, 2004). PAR-1 also plays important roles in tissue repair processes. Thrombin, produced in response to vascular injury, stimulates the proliferation and migration of endothelial cells, epithelial cells, fibroblasts, and vascular smooth muscle cells. In addition, thrombin promotes cell motility and extracellular matrix production, facilitating tissue remodeling processes (Coughlin, 2005). Activation of PAR receptors stimulates fibroblasts to secrete connective tissue growth factor (CTGF), which promotes their growth and the production of collagen and fibronectin (Chambers et al., 2000). Similarly, vascular smooth muscle cells, under the same mechanism, proliferate and produce vascular endothelial growth factor (VEGF), a key stimulator of endothelial cell growth. Platelets activated by thrombin also release VEGF (Weber and Griendling, 2004). In endothelial cells, thrombin stimulates the expression of the vascular endothelial growth factor receptor (VEGFR), enhancing the response of these cells to growth factors and promoting the repair of damaged vessels (Patterson et al., 2001).

PAR-1 also influences the blood coagulation process and the regulation of blood flow (Brass, 2003). This receptor is present on the surface of platelets (PLTs), and its interaction with thrombin leads to platelet activation (even at very low concentrations of thrombin), including changes in platelet shape, the release of granule contents, and platelet aggregation (Lapetina, 1990). This occurs because PAR-1 interacts with three types of G proteins: G12, Gq, and Gi. Through the G12 protein, the Rho kinase pathway is activated, which induces changes in platelet shape (Lapetina, 1990). The Gq protein stimulates the activity of phospholipase Cβ (PLCβ), leading to the hydrolysis of phosphatidylinositol 4,5-bisphosphate into diacylglycerol (DAG) and inositol 1,4,5-trisphosphate (IP3). IP3 causes an increase in intracellular calcium ion concentration, while DAG activates protein kinase C (PKC), which, together with the elevated calcium level, initiates processes responsible for changes in platelet shape, their aggregation, and the release of intracellular granule contents (Offermanns, 2006). The change in platelet shape results from cytoskeletal remodeling, which is regulated by myosin light chain kinase (MLCK), activated by calcium ions. The secretion process occurs through the phosphorylation of pleckstrin by PKC in the presence of calcium ions, leading to the release of platelet granule contents and the surface expression of P-selectin. The integrin αIIbβ3 is activated by PKC and calcium ions, allowing for stable fibrinogen binding and the formation of aggregates. Additionally, thrombin interacts with platelets via receptors located in the GPIb-IX-V complex. Binding to GPIbα, a component of this complex, facilitates efficient activation of PAR-1 and PAR-4 receptors by thrombin (De Cristofaro and De Candia, 2003). The regulation of blood flow via PAR receptors occurs under pathological conditions, such as inflammation or the presence of toxins. In such cases, increased protease activity in the blood and enhanced expression of genes encoding PAR receptors are observed. Thrombin, acting through PAR-1 receptors present on endothelial cells and vascular smooth muscle cells, can influence the regulation of vascular wall tension (Coughlin, 2005). The reduction of vascular tone by the endothelium is mediated by nitric oxide (NO) and prostacyclin (PGI_2_). NO is responsible for the early response, while prostacyclin plays a key role in the later phase of endothelial relaxation (Hirano, 2007).

PAR-1 may also be involved in cancer-related processes. It has been observed that in many cancerous cell types, there is an increased expression of PAR-1, which closely correlates with tumor aggressiveness (Licari and Kovacic, 2009). This observation arises from the fact that the interaction between PAR-1 and thrombin activates the Ras, PKC, and MAPK pathways, leading to the proliferation of cancer cells and enhancing their ability to progress the disease (Xie et al., 2005). A significant aspect of thrombin’s role in tumorigenesis is the formation of new blood vessels around the tumor. Upon activation, PAR receptors stimulate the expression of genes encoding growth factors such as VEGF, PDGF, TGF-β, basic fibroblast growth factor (bFGF), and the VEGF receptor (VEGFR). Increased expression of VEGFR-2 in endothelial cells is crucial for the process of angiogenesis, while the increased expression of αvβ3 in tumor cells serves as a marker of angiogenesis. Furthermore, thrombin induces the activation of matrix metalloproteinases (MMPs) secreted by endothelial cells, primarily MMP-1 and MMP-2, which degrade type IV collagen. This process, associated with the localized degradation of the basement membrane, facilitates the migration of cancer cells (Maragoudakis et al., 2000).

Additionally, PAR receptors present in the cells of the nervous system, including neurons, astrocytes, and oligodendrocytes, play a crucial role in their normal function as well as in the pathogenesis of neurodegenerative diseases. At higher concentrations of thrombin, neuronal damage and cell death occur. In Alzheimer’s disease, a reduced level of endogenous thrombin inhibitors, such as antithrombins, has been observed. The activation of the RhoA pathway through PAR-1 hydrolysis leads to the induction of apoptosis in neuronal cells (Luo et al., 2007). Increased intracellular Ca^2+^ levels, triggered by PAR-1 activation, result in the release of glutamate from astrocytes (Xi et al., 2003). This process activates the ERK 1/2 and NFκB pathways, leading to the activation of microglia. Thrombin also stimulates the inflammatory cytokine pathway, including IL-1, IL-6, TNF-α, and arachidonate (Luo et al., 2007). In neurodegenerative diseases such as Alzheimer’s and Parkinson’s, the induction of nitric oxide (NO) synthesis is observed. Activation of the ERK 1/2 pathway causes astrocyte proliferation, further confirming the role of PAR-1 activation in brain injury and neurodegeneration. Moreover, thrombin activates matrix metalloproteinase 2, which may weaken the blood-brain barrier, potentially leading to its disruption. Low thrombin concentrations may have a protective effect, safeguarding against damage caused by glutamate, β-amyloid, reactive oxygen species, and hypoglycemia.

## 4 Summary

The progression of atherosclerosis is closely linked to epigenetic changes occurring in various vascular and immune cell types. These changes include DNA methylation, histone modifications, and the activity of non-coding RNAs (primarily microRNAs), which affect the expression of genes involved in inflammation, oxidative stress, and the function of smooth muscle and endothelial cells. The goals of epigenetic therapies include endothelial protection, inflammation reduction, modulation of the VSMC phenotype, and enhancement of protective microRNA activity.

In the case of platelets, epigenetic modifications within the PAR-1 gene (F2R) – such as DNA methylation, histone modifications, and regulation by microRNAs (e.g., miR-126, miR-92a) – may potentially influence platelet activation and the progression of atherosclerosis. PAR-1 expression can also be modulated by polymorphisms such as rs2227744G>A (in the promoter region) and rs1801719 C/T (in the 3′UTR), which may interact with epigenetic mechanisms. Furthermore, other polymorphisms in regulatory regions of the PAR-1 gene (e.g., −1426 C/T, −506 I/D, IVSn-14 A/T) also affect its expression and may increase the risk of thrombosis. Understanding these relationships may contribute to the development of new diagnostic and therapeutic strategies for cardiovascular diseases.

PAR-1 (protease-activated receptor-1) is a G protein-coupled receptor primarily activated by thrombin. Its activation triggers multiple intracellular signaling pathways (including Gαq, Gαi, and Gα12/13), which regulate inflammation, hemostasis, tissue repair, and tumor progression. PAR-1 plays a central role in platelet activation, the expression of cytokines and adhesion molecules, and the stimulation of angiogenesis. In the nervous system, excessive PAR-1 activation contributes to neurodegenerative processes. Receptor regulation involves desensitization, internalization, and lysosomal degradation. Dysregulation of PAR-1 signaling is implicated in the pathogenesis of atherosclerosis, thrombosis, cancer, and neurodegenerative diseases.
